# Non-Viral Gene Delivery Systems for Treatment of Myocardial Infarction: Targeting Strategies and Cardiac Cell Modulation

**DOI:** 10.3390/pharmaceutics13091520

**Published:** 2021-09-19

**Authors:** Jieting Wang, Luying Yu, Ao Zhou, Jie Liu, Kai Wang, Ying Luo, Fang Wang

**Affiliations:** 1 Key Laboratory of Nanomedical Technology (Education Department of Fujian Province), Nanomedical Technology Research Institute, School of Pharmacy, Fujian Medical University, Fuzhou 350122, China; jietingwang@fjmu.edu.cn (J.W.); luyingyu@fjmu.edu.cn (L.Y.); aozhou@fjmu.edu.cn (A.Z.); 2 Department of Biomedical Engineering, College of Engineering, Peking University, Beijing 100871, China; kzhou@pku.edu.cn; 3 Key Laboratory of Molecular Cardiovascular Science (Ministry of Education), Department of Physiology and Pathophysiology, School of Basic Medical Sciences, Peking University, Beijing 100191, China; kai.wang88@pku.edu.cn; 4 Department of Biomedical Engineering, Tufts University, Medford, MA 02155, USA

**Keywords:** gene therapy, non-viral delivery systems, myocardial infarction, nanoparticles

## Abstract

Cardiovascular diseases (CVD) are the leading cause of morbidity and mortality worldwide. Conventional therapies involving surgery or pharmacological strategies have shown limited therapeutic effects due to a lack of cardiac tissue repair. Gene therapy has opened an avenue for the treatment of cardiac diseases through manipulating the underlying gene mechanics. Several gene therapies for cardiac diseases have been assessed in clinical trials, while the clinical translation greatly depends on the delivery technologies. Non-viral vectors are attracting much attention due to their safety and facile production compared to viral vectors. In this review, we discuss the recent progress of non-viral gene therapies for the treatment of cardiovascular diseases, with a particular focus on myocardial infarction (MI). Through a summary of delivery strategies with which to target cardiac tissue and different cardiac cells for MI treatment, this review aims to inspire new insights into the design/exploitation of non-viral delivery systems for gene cargos to promote cardiac repair/regeneration.

## 1. Introduction

Cardiovascular diseases (CVD) are the leading cause of death worldwide. During the past decade, the global number of deaths attributable to CVD increased by 12.5%, and CVD accounted for 33% of total deaths globally in 2019 [1,2]. Among the types of CVD, ischemic heart diseases (IHD) ranked first for disease burden in 2015 [3]. Myocardial infarction (MI) is a form of IHD and is usually caused by the occlusion of coronary arteries due to floating atherosclerotic plaques. Blockades in blood vessels immediately interrupt the supply of oxygen and nutrients, and if the blockades cannot be removed within 15 min, the cells in the infarct zone begin to die and release chemotactic factors that initiate acute and chronic inflammatory processes, eventually causing ventricle dilation and heart failure [4]. The therapeutic outcomes of current surgical interventions or pharmacological treatments are limited due to the failure to repair infarcted tissue [5], implying an urgent need for new, alternative strategies.

### 1.1. Clinical Trials of Gene Therapy for Heart Diseases

Gene therapies utilize the nucleic acids as drugs to introduce a new gene or alter disease-causing gene expression in cells, providing advantages such as facilitating specific manipulation, long-term therapy, the replacement of damaged cells and the potential permanent fixation of abnormal genes to eliminate diseases [6]. It was reported recently that CVD were the third frequent indication for clinical trials of gene therapy and was targeted by 5.0% of all gene therapy trials carried out globally from 1988 to 2022 [7]. The successful clinical translation of gene therapy depends on sophisticated delivery technologies to improve the stability, target affinity and internalization of nucleic acids. Viral vectors have constituted a major part of the clinical trials of cardiac gene therapies to date. As shown in Table 1, viral vectors were used to transduce the sarcoplasmic/endoplasmic reticulum calcium ATPase 2 (SERCA2a), adenylyl cyclase 6 (Ad5.hAC6), and inhibitor-1 (I-1c) genes into the myocardium to promote the function of cardiomyocytes (CMs). The Ad5.hAC6 gene moved to phase 3, while the SERCA2a gene failed in phase 2b due to ineffectiveness. To target the pathways of angiogenesis, the VEGF, VEGF-D, and FGF-4 genes were also delivered using viral vectors, while a non-viral cationic polymer carrier was used in the in vitro transfection of endothelial progenitor cells for transplantation. Two trials directly delivered SDF-1 and VEGF-165 plasmid DNA to stimulate the homing of stem cells into the myocardium and promote angiogenesis, respectively [8].

### 1.2. Non-Viral versus Viral Vectors

As viruses have essentially evolved to deliver gene cargos, they are typically very efficient and, as shown above, constitute a major part of current clinical trials for heart diseases [28,29]. However, safety concerns have greatly limited the application of viral vectors for gene delivery. The random insertion of genes into the genomes of repopulating cells via viral vectors could cause mutagenesis and oncogenesis [30]. Viral vectors may express immunogenic epitopes, inducing dangerous immune responses [31]. The lack of cell/tissue targeting has also hampered the exploitation of viral vectors, especially for in vivo gene delivery [32]. Other challenges with viral gene delivery include the limited packaging capacity of gene cargos and the high cost of vector production [33]. Non-viral materials, such as polymers and lipids, have been developed to create ‘synthetic artificial viruses’ for the delivery of genes into cells in vitro and in vivo [34]. Owing to their limitations in overcoming extra- and intracellular obstacles, these synthetic materials can exhibit poor delivery efficiency relative to viral vectors [29]. However, considering their biocompatibility, high loading capacity, facile fabrication, and potential large-scale production, non-viral vectors have shown great progress in the delivery of gene cargos, especially small interfering RNA (siRNA) molecules. In 2018, the first FDA approved small interfering RNA (siRNA) therapeutics demonstrated the promise of non-viral lipid carriers for RNA interference (RNAi) therapy [35,36]. Following this, the release of two siRNA-based drugs and the recently approved mRNA vaccine delivered through lipid nanoparticles (LNPs) further proved the potential of non-viral nanomaterials for gene therapy. The nanomaterials could be functionalized with small molecules or biomolecules to modify their physicochemical properties such as charge density, hydrophobicity, degradability, and affinity of binding to specific cells [37]. Owing to their controllable physicochemical parameters, nanomaterials are promising for targeted, sustained release, and environmentally responsive gene delivery.

### 1.3. Delivery Barriers

**Figure 1:**
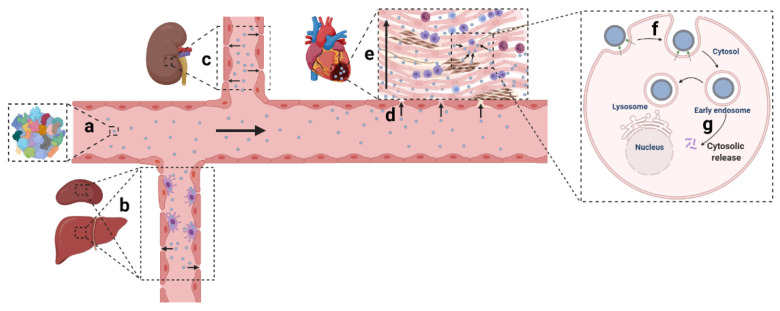
Delivery barriers of nanoparticles targeting an injured heart. (**a**) Formation of protein corona shielding the surface of nanoparticles. (**b**) Sequestration by MPS. (**c**) Filtration through the kidney. (**d**) Extravasation into the cardiac tissue. (**e**) Penetration in the tissue. (**f**) Uptake by cardiac cells. (**g**) Endosomal escape releasing cargos to cytosol.

Owing to the development of material sciences and nucleic acid chemistry, numerous non-viral nanomaterials have been created and applied in various disease circumstances. Among the 48 clinical trials involving siRNA-based therapy, most are aimed to manage cancer in tissues such as the liver, eye, and skin [38,39]. RNA (including siRNA, microRNA (miRNA), messenger RNA (mRNA), short hairpin RNA (shRNA)) therapeutics targeting the cardiac tissue, and CVD still remain to be developed. Ingenious delivery platforms were urgently needed to broaden clinical exploitations of gene therapy in CVD. Non-viral nanoparticulate systems designed for gene delivery generally encounter systemic and local hurdles: (1) the mononuclear phagocytic system (MPS) barrier, kidney filtration, and protein corona shielding in circulation; (2) extravasation, penetration, and retention in the tissue; (3) internalization and release in cells (Figure 1) [40,41].

Once exposed in blood, nanoparticles are usually coated by various circulatory proteins which mask their surface modification and change the biodistribution of nanoparticles [42,43]. With a specialized vessel structure consisting of fenestrated endothelia, abundant macrophages lying on the lumen side of blood vessels and a lower nanoparticle velocity (about 1000-fold) in the liver, the MPS was reported to clear more than 99% of nanoparticles from circulation [44,45,46,47,48]. If the diameters of the nanoparticles are smaller than the filtration slits (about 4~6 nm) in the kidneys, the nanoparticles would be filtered out and eliminated into the urine [49,50]. The entry of nanoparticles to an injured heart is suggested to result from passive movement through the leaky vasculature, similar to the enhanced perfusion and retention (EPR) effect found in tumors [51,52]. In cardiac tissue, nanodelivery systems usually need to cover the injured area (about 5~50% of the heart section), which is impeded by the difficulty in the penetration of nanoparticles due to the complex interactions (e.g., electronic interactions) of nanoparticles with the compact extracellular matrix. It has been reported that particles administered intramyocardially can only diffuse approximately 1–2 mm away from the injections site [53,54]. In a tumor, gold nanoparticles with or without targeted peptides could not penetrate further than 15 µm from the walls of blood vessels [55]. Nanoparticles mainly resided in the border zone between the injury and healthy area, whether injected locally or systemically at early or later stages of the heart injury [53,56,57]. Local injection into the myocardium was supposed to improve the contents of drugs in tissue, while mechanical extrusion out of the beating heart and rapid diffusion into the adjacent tissue areas always led to low retention for nanotherapeutics relative to that for other tissues [58]. The retention time of nanoparticles was generally <72 h in cases of local or systemic injection at 1 day or 7 days post-infarction [59,60]. At the internalization level, cardiomyocytes were relatively hard to transfect due to their non-phagocytic nature. In cytosol, gene cargos needed to be released into the cytoplasm or nucleus to perform gene regulation. It was reported that the release of siRNA occurred invariably from maturing endosomes within about 5~15 min with endosomal escape efficiency of about 3.7%, and about 2000 cytosolic siRNA molecules were needed for each cell to achieve efficient gene silencing [61]. Adding functional moieties or amplifying the ‘proton sponge effect’ to enhance the endosomal escape of gene cargos may be valuable for efficient release in the cytosol [62,63].

This review aimed to summarize the current progress in strategies combating the physiological barriers faced by non-viral delivery systems for cardiac transfection or targeting. Considering the recent knowledge on the repair/regenerative molecular mechanisms in MI, applications of non-viral carriers to target different cardiac cells were reviewed to inspire novel designs of delivery systems to manipulate these gene targets.

## 2. Non-Viral Delivery Systems with High Transfection Efficiency for Cardiac Cells

### 2.1. General Construciton Strategies

The nature of the cargo determines the basic requirements for delivery systems. Instability in the presence of nucleases is one of the major concerns for gene-based drugs, especially for small RNA molecules, which are more sensitive to enzymatic degradation. The anionic phosphate backbone is another obstacle to be overcome, as the repulsive electrostatic interaction between nucleic acids and cell membranes impedes gene molecules’ free diffusion into cells. In addition, the short half-lives of gene drugs (e.g., 5 min for siRNA molecules following intravenous injection) also demonstrate the need for a delivery system to improve the pharmacokinetic properties [64,65]. 

To meet the requirements mentioned above, various types of delivery vehicles—mainly polymeric materials and liposomes—have been studied for compacting or loading gene cargos into nanoscale particles for delivery in cardiac tissue. The particles protect the integrity of nucleic acids in the presence of nucleases and also optimize their pharmacokinetics by altering their sizes and surface properties. Reineke et al. designed a series of poly(glycoamidoamine) (PGAA) materials by incorporating a carbohydrate comonomer within a PEI-like backbone [66,67]. All the PGAAs showed minimal cytotoxicity, and the tartarate-incorporated T4 glycopolymer complexed with NF-κB oligodeoxynucleotide (ODN) decoys showed 87% penetration of the myocardium in mouse hearts and a nearly complete reduction in Cox-2—a well-known NF-κB dependent gene in the heart—with a dose of 10.0 μg [68]. Micelles, liposomes, and silica particles were also used as delivery systems in the heart [60,69,70,71].

### 2.2. Particle Surface Modification to Enhance Transfection Efficiency

To improve the internalization efficiency in cardiac cells, the modification of basic vehicles using additional components has become a common strategy. Cell penetrating peptides (CPPs) are a major category of ligands used for this purpose, especially TAT and oligo-arginine (R9). The methods for incorporating CPPs into delivery vehicles are diverse, such as complexing with gene cargos [72], conjugating to DNA or RNA molecules [73], modifying the surface or scaffolds of liposomes or polymers [74,75], and decorating other complicated systems [76]. All the results show that, compared to unmodified vehicles, CPPs did enhance the internalization of delivery systems in CMs, while simple cationic particles, such as those made from polyamidoamine (PAMAM) and PEI, failed to achieve efficient delivery [77,78]. It is also interesting to note that the modification of R9 showed better performance than that of TAT in transfecting CMs in vitro and, most likely, delivering gene cargos in cardiac tissue in vivo [78]. Youngsook Lee et al. also demonstrated the effectiveness of arginine-grafted polymer for plasmid human erythropoietin gene delivery in a rat MI model [79]. It is possible that the sequence and structure of pure oligo-arginine satisfy the preference of CMs, making it a potent element with which to construct delivery systems for hearts. The transferrin receptor, also called CD71, mediates the transport of ferric ions into cells via transferrin (Tf). The group of Hirokazu Matsumoto found that the siRNA-conjugated anti-CD71 Fab’ fragment resulted in efficient gene silencing in the liver, heart and calf muscles in healthy mice after intravenous administration, and the silencing of myostatin in a peripheral artery disease (PAD) model of mice with femoral artery ligation led to the recovery of leg functions [80]. Since CMs are rich in transferrin receptors involved in myoglobin synthesis [81], the anti-CD71 antibody could be a potential ligand of nanoparticles for the efficient delivery of gene cargos to the CMs.

To guide delivery systems into a certain type of cell, cell-type-specific ligands have been incorporated into vehicles, including carbohydrates, peptides, proteins, etc. GlcNAc is a saccharide ligand specific to CMs screened out from a carbohydrate library, and its conjugation facilitated the successful delivery of a liposome system and a polyketal system into CMs [82,83]. As for peptide ligands, at least three candidates have been identified from phage display. PCM is a ligand that targets primary CMs [84], and Bull et al. used this peptide in their polymeric systems to deliver siRNA to CMs in vitro [73,74]. Molecules that can recognize the membrane proteins of CMs constitute another type of potential ligand. For example, PGE-2-conjugated siRNA was used in Bull’s study to induce receptor-mediated endocytosis in H9C2 cells [85]. Delivery systems guided by specific ligands do possess superior binding/internalization abilities in CMs compared to unmodified systems, while CPP-guided delivery systems still have a higher delivery efficiency in general. Consequently, combining the two types of ligands may become a useful method for developing delivery systems in cardiac tissue, which, in fact, has shown some synergic effects in transfecting CMs in vitro and in vivo [56,73,74,86].

## 3. Non-Viral Delivery Systems Capable of Targeting Cardiac Tissue

**Figure 2:**
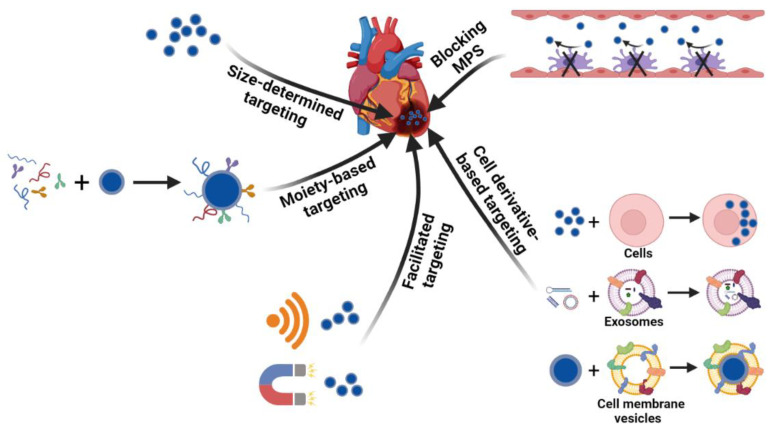
Targeting strategies of non-viral delivery systems for an injured heart.

Targeted delivery to the heart through systemic administration is a minimally invasive treatment method. Strategies developed for targeting other tissues could be utilized for the heart in which vectors are usually added with targeting components, facilitated by external sources, integrated with biological systems possessing innate natural properties or enhanced targeting via blocking MPS (Figure 2). The targeting methodologies for a damaged heart are summarized in this section.

### 3.1. Size-Determined Targeting

V.J. Caride et al. first demonstrated the capability of systemically injected liposome nanoparticles to accumulate in myocardium in 1977 [52,87]. Nanoparticles of a suitable size could accumulate in the infarcted cardiac tissue. For example, Uskov et al. demonstrated an increased accumulation of silica nanoparticles with diameters of 6–13 nm in cardiac tissue after ischemic injury compared to without injury [70,88]. Several studies have shown that PEGylated liposomes and micelles or PEG-modified polystyrene nanoparticles with diameters between 15 and 400 nm showed similar extents of accumulation, while particles larger than 0.5 μm showed decreased deposition in the infarcted heart [69,89,90,91]. The mechanism behind the cardiac accumulation needs more investigation, despite some research having claimed that the damaged heart accumulates nanoparticles via an EPR-like mechanism due to the leaky vasculature [51,52].

### 3.2. Moiety-Based Targeting

In addition to the size affecting the accumulation, nanoparticles conjugated with targeting moieties have shown enhanced final delivery efficiency. The targeting ligands recognize cardiac tissue through a specific interaction with certain receptors or markers in the myocardium and promote the accumulation of the delivery vehicles in the heart. Theoretically, nanoparticles can be modified to target all types of cells or special components in the extracellular environment in the heart. Cardiomyocytes are the most studied targets. Ligands targeted to cardiomyocytes have generally been found by phage display. Through in vivo phage display, several peptides such as CSTSMLKAC, CRPPR, and I-1 were screened out for cardiomyocyte targeting, all of which show an increase in accumulation and efficiency in the injured heart compared to nanoparticles without peptide conjugation [57,92,93]. Another candidate, called ischemic myocardium-targeted peptide (IMTP), was conjugated to a cystamine bisacrylamide-diamino hexane polymer with modification of the R9 peptide (IMTP-CD-9R), and the intravenous injection of IMTP-CD-9R/HO-1 plasmid complexes induced high HO-1 expression in ischemic injured left ventricle tissue, demonstrating the targeting efficacy of IMTP [86]. Instead of peptides screened from phage display, Kohane et al. utilized an Ang II sequence on liposome vehicles to recognize the Ang II receptors on cardiac cells, which resulted in a higher accumulation of liposomes than the scrambled sequence [94]. The myosin expressed by damaged cardiomyocytes could also be used as a target for nanoparticles [56,89].

Other cell types, including endothelial cells and macrophages, could also be targeted by nanoparticles. The anti-P-selectin ligand and RGD peptide were used for endothelial cell targeting to promote angiogenesis in myocardial infarction model in rats [95,96]. Liposomes modified with phosphatidylserine (PS) were designed for the modulation of cardiac macrophages to a reparative state at a predetermined time after MI, which promoted angiogenesis and prevented ventricular dilation and remodeling [97]. In addition to cells, the extracellular environment could also be targeted via ligands. For instance, the group of Michael E. Davis designed Hoechst conjugated insulin-like growth factor-1 (IGF-1) nanoparticles to target DNA released from dying cells in an infarcted heart [98]. The conjugation of Hoechst enhanced the retention of IGF-1 in the infarct area and attenuated MI-induced cardiac dysfunction and fibrosis. Other pathological characteristics such as the high abundance of fibrin or leakage of cardiac troponin I (cTnI) from dead cells can also be targeted. Zheyong Huang et al. utilized CREKA peptide which was screened from an in vivo-phage display for fibrin targeting [99]. The CREKA-conjugated PEG-PLA nanoparticles showed enhanced accumulation and retention in the injured heart compared to particles without conjugation. The Haisheng Peng group modified liposomes with anti-cTnI antibody to deliver anti-miR-1 antisense oligonucleotides (AMO-1) to the ischemic myocardium in rats, which downregulated miR-1 expression and showed functional benefits [100].

### 3.3. Facilitated Targeting

Facilitated targeting utilizes external sources to lead delivery systems to reach the target sites. Ultrasound-targeted microbubble destruction (UTMD) is the most commonly used method in this area. Ultrasound energy is able to enhance the local vascular permeability and simultaneously destruct the gene- or protein-loaded microbubbles into pieces to release the cargos on site. The delivery efficiency is dependent on the time and ultrasound energy. Li Zhang et al. utilized UTMD to locally deliver PHD2 shRNA to an ischemic heart for the treatment of MI. The silencing of PHD2 reduced the expression of HIF-1α, VEGF and bFGF, which promoted neovascularization, reduced the infarct size and improved the cardiac functions [101]. Although quite a few studies have proven the efficacy of UTMD in animal models of MI or IR [102,103,104,105,106], concerns still exist regarding the translation to human applications, such as the potential risk of tissue damage. Another method is to use a magnetic field to guide magnetic-particle-incorporated systems to the heart. For instance, Ma et al. successfully used magnetic nanobead/adenoviral vector complexes to induce VEGF expression in rat hearts with the facilitation of a magnetic field [107]. Compared to UTMD, this strategy avoids tissue damage and is easier to transform to human applications, but it needs to be further developed to carry other categories of cargos. 

### 3.4. Cell Derivative-Based Targeting

As natural materials, cells or cell derivatives hold promise for targeted delivery due to advantages such as high biocompatibility, homing capability, and inherent therapeutic functions for the treatment of diseases. Circulating cells such as red blood cells, stem cells, and leukocytes were recently leveraged for drug delivery due to their long circulation times and tissue-homing properties in vivo [108,109]. A recent study by Molly M. Stevens group showed that neutrophils loaded with liposomes ex vivo could transport nanoparticles to inflamed skeletal muscle and ischemic hearts in mice, demonstrating neutrophils to be suitable carriers of nanoparticles to target an injured heart due to their inherent homing to inflammatory tissue [110]. To target migrating monocytes in circulation to mediate cardiac targeting of nanoparticles, Patrick C. H. Hsieh’s group developed a platelet-like proteoliposome (PLP) in which liposomes were incorporated with proteins isolated from platelets [111]. The specificity of the PLP was proven by the strong binding affinity for monocytes but not endothelial cells in vitro. Following intravenous injection, the PLP showed higher accumulation in the heart after 72 h of injection, and the loading of the anti-inflammatory drug resulted in a better therapeutic outcome than the liposomes without protein incorporation. Exosomes can be genetically engineered with targeted peptides for cardiac targeting. Ji-Young Kang et al. developed a cardiac-targeting peptide-modified exosome derived from the transgenic engineering of HEK293 cells. The co-delivery of curcumin and miR-144-3p in this carrier showed cardioprotective effects both in vitro and in vivo for MI treatment [112]. Cell membranes are another class of naturally derived materials possessing innate properties inherited from source cells. By mimicking the ability of mesenchymal stem cells (MSC) to target an injured heart, Chi Yao et al. utilized MSC membranes to coat miR-21-loaded silica nanoparticles, which enhanced the accumulation and sustainable release of miR-21 in the damaged heart, resulting in the inhibition of cardiomyocyte apoptosis and preservation of cardiac function against MI injury [113].

### 3.5. Blocking MPS

As the major hurdle for systemic targeted delivery is the MPS [48,114], previous studies have usually prevented nanoparticles from interacting with immune cells to evade the MPS by coating the nanoparticles with stealth shells or allowing them to ‘hitchhike’ on the red blood cells (RBCs) or other circulatory cells. Recent studies have offered a different strategy—blocking the MPS. The group of Petr I. Nikitin utilized the RBC antibody (IgG2a-34-3C) to bind circulatory erythrocytes to stimulate erythrophagocytosis, which intensified the clearance of the organism’s own intact blood cells. The ingestion of RBCs transiently blocked the phagocytosis of nanoparticles by the MPS and enhanced the circulation time and tumor accumulation of nanoparticles up to about 32-fold and at least 14-fold, respectively, compared to the direct injection of nanoparticles without the MPS-cytoblockade [115]. The MPS-blocking strategy could also be utilized for cardiac targeting. Another study showed that exosomes loaded with siClathrin silenced the expression of Clathrin in macrophages, which inhibited the endocytosis of macrophages and blocked the clearance of later injected exosomes by the MPS. Compared to the direct administration of therapeutic exosomes encapsulated with miR-21, the prior injection of exosomes blocking the MPS enhanced miR-21 accumulation in the heart by two-fold and produced a much better therapeutic effect on cardiac function in the doxorubicin-induced cardiotoxicity mouse model [116].

## 4. Modulating Different Cells for MI Treatment

**Figure 3:**
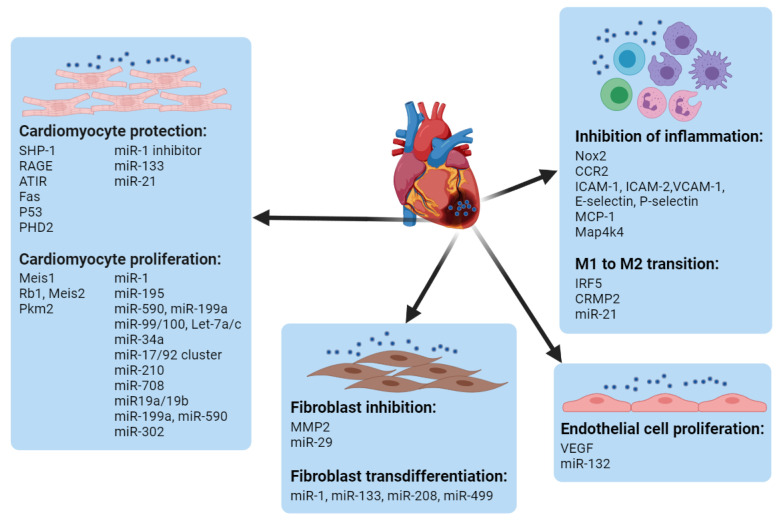
Genes or miRNAs in different cardiac cells that have been utilized as therapeutic targets in non-viral delivery systems for cardiac repair/regeneration.

The heart is mainly composed of cardiomyocytes, endothelial cells, fibroblasts, and tissue resident macrophages, which constitute about 33%, 40%, 11~27%, and 4~5% of the total cardiac cells, respectively [117,118,119,120,121], all of which play important roles in cardiac function. Based on the understanding of molecular mechanisms during the MI, nucleic-acid delivery systems are being developed to target different cells in the heart for cardiac repair/regeneration. Non-viral systems can be exploited to deliver gene cargos to not only one type of cell but also different cells concurrently or successively based on the demands of modulation at different stages of injury. For instance, the group of Kim utilized the PEI1.8-DA carrier to simultaneously deliver siSHP-1 and VEGF plasmids, which target the cardiomyocytes and endothelial cells, respectively, for the treatment of MI in rats [122]. Jinli Wang et al. constructed miR-101a-loaded MSC exosomes for MI repair. Here, miR-101a was utilized for the inhibition of fibrosis, and the MSC exosomes were leveraged for the polarization of macrophages from M1 to M2. The therapeutic co-effects from the gene cargo and the carrier increased cardiac function in the MI model of mice [123]. Using ethanolamine (EA)-modified poly(glycidyl methacrylate) (PGEA) to fabricate heparin-cored nanoparticles (Hep@PGEA), Jing-Jun Nie et al. sequentially delivered miR-499 and VEGF DNA for the inhibition of cardiomyocyte apoptosis in the early stages of MI and to promote angiogenesis at later stages. This chronological treatment restored the heart’s function and suppressed cardiac hypertrophy [124]. Given the significance of CMs, endothelial cells, immune cells and fibroblasts, non-viral delivery systems leveraged to manipulate gene targets in different cardiac cells are summarized in this section (Figure 3).

### 4.1. Cardiomyocyte Protection and Proliferation

CMs constitute approximately one-third of the total cell number in the heart while constituting approximately two-thirds of the volume of the heart [120]. When injury occurs, millions of cardiomyocytes die within a few hours. A direct way to mend the injured heart would be to protect the CMs from death or promote the proliferation of CMs to counter the loss.

A facial amphipathic deoxycholic-acid-modified PEI (1.8 kDa) (PEI1.8-DA) conjugate was synthesized by Kim et al. to deliver SHP-1 siRNA in a rat ischemia/reperfusion (I/R) model [125]. The treatment decreased elevated SHP-1 expression to the normal level and significantly reduced the cardiomyocyte apoptosis and infarct size to a 7.7% apoptosis index and 5.4%, respectively. The delivery of siRAGE molecules using the same vector also decreased the apoptosis of CMs and depressed the immune responses in MI in rats [126]. After modifying the cationic polymer of poly(CBA.DAH) (PCD) with PGE2 or a targeting peptide (WLSEAGPVVTVRALRGTGSW, PCM) that was screened from phage display, Fas siRNA could be efficiently delivered into CMs, which reduced the apoptosis under hypoxic conditions in in vitro [85]. The PCM peptide could also enhance the targeting ability of stearic acid carboxymethyl chitosan nanoparticles loaded with P53 siRNA for cardiomyocytes and inhibit the hypertrophy of cardiomyocytes in vivo after intravenous administration [127]. A PAMAM-based system was utilized to locally deliver AT1R siRNA molecules to CMs in a mouse MI model, and the reduced infarct size and improvement in cardiac functions demonstrated the efficiency of the carrier [78]. PHD2 is another factor related to apoptosis. Kai Zhu et al. developed an arginine-terminated generation 4 PAMAM system to deliver siPHD2 to MSC. The transplantation of genetically modified MSC to the MI site enhanced the survival of MSC and CMs, decreased the scar size and interstitial fibrosis, promoted angiogenesis, and improved the cardiac functions [128].

miRNAs are endogenous post-transcriptional regulatory molecules that could be involved in the regulation of cardiomyocyte apoptosis. To systemically deliver the miR-1 inhibitor to protect CMs, the group of Bin He designed a dendrigraft poly-L-lysine (DGL) vector conjugated with an AT1-targeting peptide. The efficient delivery resulted in a reduction in infarct size in an MI model [129]. However, the small thrombi in the capillaries could hamper the delivery of the nano-vector, for which they modified the surface of the nano-vector with low-molecular-weight heparin (LMWH). The heparin decoration further enhanced the accumulation of the miR-1 inhibitor in the injured heart and improved the therapeutic outcomes [130]. Bixi Sun et al. utilized arginine–glycine–aspartic acid tripeptide (RGD) in polyethylene glycol (PEG)–polylactic acid (PLA) nanoparticles for the targeted delivery of miR-133 to an injured heart. The improved accumulation of miR-133 reduced the apoptosis of CMs, decreased the levels of factors associated with MI, and alleviated cardiac histopathological changes [131]. miR-21 is another target related to CM apoptosis. To efficiently deliver miR-21, Minghui Li et al. fabricated a liposome vector modified with the cardiac troponin T (cTnT) antibody capable of systemically targeting the ischemic myocardium. The vector increased the content of miR-21 in the heart, which maintained the viability of CMs, decreased the infarct size and improved the cardiac function after acute MI [132]. Given that exosomes are innate carriers of nucleic acids and proteins for cell–cell communication, exosomes containing miR-21, which were derived from cells overexpressing miR-21 or prepared by encapsulation, were exploited for the treatment of MI. After intramyocardial administration, the apoptosis of CMs and endothelial cells was prevented and the cardiac dysfunction was attenuated [133,134]. Other miRNAs in curative exosomes, such as miR-148a, miR-1271-5p, miR-19a, and miR-150-5p, were found to be critical for the treatment of MI, demonstrating the potential of these molecules to be targets of non-viral delivery systems for cardiac recovery [135,136,137,138].

CMs generally stop proliferating and grow further through the hypertrophy of existing cells after birth in mammals. Nevertheless, there is evidence of human CM regeneration [139]. The Hesham A. Sadek’s group found that Meis1 could inhibit the proliferation of CMs, and the transfection of Meis1 siRNA via the Lipofectamine commercial carrier promoted the proliferation of CMs in vitro, suggesting that the Meis 1 could be a potential target for the modulation of cardiac regeneration [140]. Rb1 and Meis2 are negative regulators of the cell cycle and the differentiation state in CMs, respectively. Perwez Alam et al. delivered a Rb1 and Meis2 siRNA cocktail using commercialized Lipofectamine RNAiMAX to promote the proliferation of CMs, with about 30% efficiency in vitro. The siRNA cocktail loaded in a hydrogel system also promoted the proliferation of CMs in vivo, which improved cardiac function after intramyocardial injection into a heart with MI [141]. Pkm2 (pyruvate kinase muscle isoenzyme) was reported to be related to the cell-cycle re-entry of CMs, and the intramyocardial injection of modified Pkm2 mRNA increased CM division, cardiac function, and long-term survival in cases of acute or chronic myocardial infarction in mice [142]. Other gene targets such as cyclin D2, PDK4 (pyruvate dehydrogenase kinase 4) and long noncoding RNA CPR (cardiomyocyte proliferation regulator) were also found to be correlated to cardiomyocyte proliferation, but non-viral systems for manipulating these genes remain to be developed [143,144,145].

In recent years, it has been found that miRNAs such as miR-1, miR-195, miR-590, miR-99/100, miR-17/92, miR-199, miR-210, and miR-34a could be relevant in the proliferation of CMs [146,147,148,149,150,151,152,153], indicating the potential of miRNA molecules to activate endogenous cardiac regeneration. Yuxin Wang et al. transfected MSC to express cTnI-targeted short peptides on the surface to derive cTnI-targeted exosomes for the systemic delivery of miR-590-3p, which could be endocytosed by CMs in the injured heart and promoted cardiomyocyte proliferation, restoring cardiac function following MI [154]. Shengqiong Deng et al. found that miR-708 was abundant in neonatal mice, while the expression level was reduced in adult rat hearts. After the intravenous administration of an miR-708 mimic using the commercial neutral lipid carrier RAN-LANCEr II to CMs under hypoxia or isoproterenol treatment, the proliferation of CMs increased and the cardiac regeneration and function improved. The inhibition of MAPK14 by miR-708 was suggested to contribute to cell-cycle arrest in CMs [155]. Dazhi Wang et al. demonstrated the therapeutic potential of the miR-17/92 cluster in myocardial infarction by intramyocardially or systemically delivering the miR-19a/19b mimic complexed with a commercial lipid carrier, which promoted the proliferation of cardiomyocytes and showed cardiac-protective effects for 14 weeks [156]. Mauro Giacca’s group also intracardially administered the hsa-miR-199a-3p and hsa-miR-590-3p mimics into mice with MI using a commercial lipid carrier and demonstrated the efficiency of the synthetic miRNA-lipid formulation in stimulating cardiac repair and restoring cardiac function [157]. The group of Kai Li incorporated miR-199a-3p nanoparticles composed of cell penetration peptide, PFBT, and lipid-conjugated miRNA into a protein-engineered hydrogel for the sustained release of miRNA to improve cardiac function following MI [58]. By utilizing an injectable hyaluronic-acid hydrogel, the group of Jason A. Burdick demonstrated that the local and sustainable delivery of miR-302 to an infarcted heart could enhance the proliferation of cardiomyocytes in the border zone and ameliorate the cardiac function [158]. Other miRNA molecules, such as miR-1825 and miR-294, were also found relating to cardiomyocyte proliferation, which was demonstrated via viral vectors [159,160]. The potentials of non-viral systems to deliver these molecules are worthy of further investigation.

### 4.2. Endothelial Cell Proliferation and Revascularization

Another direct way to treat an ischemic heart is to increase the supply of blood by promoting revascularization, which may need to be controlled for a specific duration. To sustainably deliver VEGF DNA into the heart, Arghya Paul et al. developed a hydrogel system in which DNA was complexed with PEI-functionalized graphene oxide (GO) nanosheets and incorporated into a low-modulus methacrylated gelatin (GelMA) hydrogel. Local administration increased the capillary density at the injected peri-infarct region and reduced the scar area, which improved cardiac function in a rat MI model [161]. However, Lior Zangi et al. claimed that the vessels formed in response to the long-term expression of VEGF-A delivered via DNA were highly permeable, and the leaky vessels would result in edema. Compared to the poor survival observed with VEGF DNA administration in the first month following MI, they found that the transient expression of VEGF-A (within 2~3 days) via the delivery of modified mRNA (modRNA) using a commercial transfection reagent (Lipofectamine RNAiMAX) could induce functional vessels, attenuate cardiac dysfunction, and improve the survival [162]. The transient induction of gene expression in an MI heart was also achieved through the delivery of modRNA using a lipidoid vector. Irene C. Turnbull et al. demonstrated the efficiency of lipidoids for delivering eGFP modRNA in the hearts of rats and pigs with MI. The expression of eGFP was controlled within 2~3 days, and the delivery via lipidoid was about 60-fold higher than that via naked modRNA at 20 h after intramyocardial injection [163]. It indicated the potential of the lipidoid delivery platform for modRNA to induce the transient expression of genes, such as VEGF, for MI treatment. To target endothelial cells, the group of Daniel G. Anderson developed a 7C1 polymer to encapsulate siRNA that could specifically silence Tie2 in the endothelial cells in all organs, including the heart in primates, demonstrating the potential of the 7C1 polymer for RNAi therapy to target cardiac endothelial cells [164]. The CD151 gene delivered using the rAAV viral vector intramyocardially could promote neovascularization in MI [165], demonstrating the potential of the CD151 gene as a therapeutic target for non-viral delivery systems.

miRNA molecules can also regulate endothelial cell proliferation. Teng Ma et al. encapsulated miR-132 into exosomes derived from MSC. After the local injection of the miR-132-loaded exosomes to the infarcted heart, neovascularization in the peri-infarct zone was enhanced, and the heart’s functions were preserved [166]. miR-24 was found to be upregulated in endothelial cells after MI induction, for which the group of Costanza Emanueli locally delivered an miR-24 decoy using an adenovirus to inhibit miR-24 expression, which increased angiogenesis and blood perfusion in the peri-infarct myocardium, reduced the infarct size, induced fibroblast apoptosis, and improved overall cardiac function [167].

### 4.3. Immune Cell Modulation

Immune cells play significant roles in the modulation of cardiac repair by affecting the functions of cardiomyocytes, endothelial cells, and fibroblasts [168]. The innate immune cells, including macrophages and neutrophils, are usually heterogeneous and plastic and can be roughly divided into pro-inflammatory type 1 and anti-inflammatory type 2, both of which are necessary for cardiac repair [169,170,171,172]. The crosstalk between innate and adaptive immune cells, e.g., T cells, is also critical for the mending of an injured heart [173,174,175]. Given the complex and multiple roles of immune cells, the modulation of immune systems may require precise spatial and temporal regulation.

One way to modulate immune cells is to inhibit the infiltration of these cells to prevent inflammation-induced injury in the heart. The occurrence of MI increases the level of Nox2, which is mainly expressed in inflammatory cells, and the silencing of Nox2 inhibits the release of inflammatory cytokines. Michael E. Davis’ group developed a polyketal system to load siNox2 [176]. After the intramyocardial administration of nanoparticles and chloroquine, the release of inflammatory factors, the death of cardiomyocytes and the formation of fibroblasts were all decreased, and the cardiac functions were improved. To target circulatory monocytes and inhibit their recruitment into the heart after MI, the group of Daniel Anderson utilized the lipidoid system to encapsulate CCR2 siRNA to abrogate the inflammatory stage and promote cardiac repair [177,178]. In another system, a lipid vector based on PEI with a molecular weight of 600 was designed for the co-delivery of multi-siRNAs silencing ICAM-1, ICAM-2, VCAM-1, E-selectin, and P-selectin, which inhibited the adhesion and accumulation of inflammatory cells in the heart, inhibiting the inflammatory stage of cardiac repair [179]. By screening the same material library and adding the optimized polyethylene glycol (PEG)–lipid conjugate to the formulation of siRNA nanoparticles, a hematopoietic niche-targeting vector was developed. The silencing of MCP-1 after the intravenous injection of siMCP-1 nanoparticles inhibited the release of leukocytes from bone marrow and preserved the cardiac function after MI [180]. Macrophages can innately phagocytose large particles, for which 2–4-micrometer-sized microparticles with glucan shells were fabricated to encapsulate siRNA molecules targeting the Map4k4 gene of macrophages. After intramyocardial injection, the inflammatory responses within the infarct were dampened, demonstrating the efficacy of immunomodulation in MI [181].

Instead of reducing the number of inflammatory cells in the injured heart, transitioning from the inflammatory phenotype to the reparative phenotype is another promising strategy. Daniel Anderson et al. delivered IRF5 siRNA to macrophages utilizing a lipidoid system, which transformed the M1 type into the M2 type to promote the reparative stage of cardiac repair and improve the cardiac functions [182]. CRMP2 (collapsin response mediator protein-2) also participates in the transition from M1 to M2, and the delivery of CRMP2 siRNA also has therapeutic benefits for MI. Long-Shu Zhou et al. utilized a lipidoid system containing cationic lipid C12-200, disteroylphosphatidyl choline, cholesterol and PEG–DMG to deliver siCRMP2, which switched the macrophages from the M1 to M2 phenotype in vitro and in vivo. The intravenous injection of nanoparticles resulted in a marked decrease in inflammation and fibrosis and significant attenuation of post-MI heart failure and mortality [183]. Interestingly, in addition to relating to cardiomyocyte protection as described before, miR-21 overexpression in macrophages could also resolve inflammation [184]. Tzlil Bejerano et al. developed a nanoparticulate system co-assembled from miR-21, Ca^2+^ and hyaluronan sulfate (HAS) to intravenously transfect macrophages in the infarct zone, which switched the phenotype of macrophages from pro-inflammatory to reparative, promoted angiogenesis, and reduced hypertrophy, fibrosis, and cell apoptosis in the remote myocardium [185].

### 4.4. Fibroblast Inhibition and Reprogramming

The formation of fibroblasts occurs in the later stage of MI, which hampers the function of the heart. After MI, matrix metalloproteinase 2 (MMP2) is overexpressed, mainly in fibroblasts, by as much as 3000-fold in infarct tissue by 8 weeks [186,187]. The group of Jason A. Burdick developed an MMP-responsive hydrogel loaded with siMMP2 to silence MMP expression in cardiac fibroblasts in vitro. The intramyocardial injection of the shear-thinning hydrogel increased the infarct thickness and improved the cardiac functions in an MI model [188]. Depressing the fibroblast stage would have benefits for repair following MI, for which purpose Rui-Quan Li et al. developed a star PGMA cationic polymer to load miR-29 to inhibit the formation of fibroblasts. The polymer facilitated the systemic targeting of miR-29 to the injured heart, which reduced the fibrosis and improved the cardiac function following MI [189].

The capability of fibroblasts to reprogram to CMs provides new opportunities for cardiac remodeling. Li Wang et al. claimed that the transdifferentiation of fibroblasts to CMs correlated with the balanced expression of the reprogramming factors Gata4 (G), Mef2c (M), and Tbx5 (T). After constructing the genes into a retrovirus vector in the splicing order of MGT, the transfection resulted in a higher protein level for M and lower levels of G and T. It significantly enhanced the reprogramming efficiency compared to separate G, M, and T transduction. The optimal transdifferentiation efficiency was about 10~15% in vitro [190]. miRNAs could also contribute to the reprogramming of fibroblasts to CMs. Tilanthi M. Jayawardena et al. screened a combination of miRNAs, including miR-1, miR-133, miR-208, and miR-499, to mediate the transdifferentiation of fibroblasts to CMs, realizing an efficiency of about 1.5~7.7%, which could be further enhanced to about 28% via additional treatment with JAK inhibitor I in vitro. A combination constructed in a lentiviral vector was intramyocardially injected to the heart, and approximately 1% of the cardiomyocytes were detected as derived from fibroblasts following 4 weeks of MI [191]. Lei Yang et al. utilized branched polyethyleneimine (BP)-coated nitrogen-enriched carbon dots to load this microRNA combination (miR-1, miR-133, miR-208, and miR-499). The transdifferentiation efficiency in vitro was determined via morphological observation under a microscope, and about 100 fibroblasts could transdifferentiate into CMs per imaging field, increasing by 0.5-fold compared to the PEI vector. The in vivo transdifferentiation efficiency was not quantified, but the markers of reprogramming were identified, the fibrotic area was reduced, and the thickness of the infarct was increased after the intramyocardial treatment of nanoparticles [192]. It was interesting that the fibroblasts could transdifferentiate via small chemical molecules instead of the genetic method. Nan Cao et al. discovered nine small chemical molecules (9C) capable of transdifferentiating fibroblasts to CMs with an efficiency of about 6.6% in vitro. The 9C-treated fibroblasts were compatible with the diseased-heart environment and could further mature into CMs in the infarcted hearts of mice [193].

## 5. Conclusions and Prospects

Although the recent development of nucleic-acid chemistry has made great progress in improving stability, reducing immunogenicity, and even facilitating self-delivery, delivery systems might still be necessary, especially for the systemic targeting of gene cargos [194,195]. With the development of material sciences, non-viral systems have shown promise. The unraveling of the molecular mechanisms behind cardiac damage and repair provides more targets to be manipulated. Gene targets or miRNA molecules that could directly replace damaged and lost cardiomyocytes via re-entry, dedifferentiation, or transdifferentiation offered new opportunities for cardiac regeneration. However, the current non-viral delivery carriers utilized in the primary discoveries of mechanisms are mostly commercial lipid carriers, and some gene targets remain to be utilized for therapy. More non-viral systems need to be developed for applications in cardiac regeneration.

Knowledge about delivery mechanisms forms the basis of designs for delivery strategies. The entry of nanoparticles to injured tissue has conventionally been considered to occur via the EPR effect. However, recent studies have found that nanoparticles can enter tumors through trans-endothelial pathways or utilize leukocytes, especially neutrophils and macrophages, as mediators for entry [196,197,198]. More technologies, e.g., real-time in vivo imaging with high resolution, may be needed to uncover the delivery process for nanoparticles. Other mechanisms of nanoparticles’ interaction with the biological surroundings, including molecules and cells in circulation and target tissue, also require further elucidation. Furthermore, the safety of nanomaterials or vectors is another concern to consider in the development of delivery systems, especially for cardiac tissue. The components, shape, and size could all contribute to the toxicity of nanoparticles via multiple pathways, such as the generation of reactive oxygen species (ROS), disruption of compartments. and activation of immune reactions [199,200]. Strategies for reducing the toxicity of nanoparticles need to be developed, and the bioactivities of the nanomaterials themselves should be taken into consideration during the design of delivery systems.

The ultimate goal of delivery systems includes active targeting, specific recognition, and precise spatial-temporal responses. Design strategies that leverage novel materials based on the deeper understanding of delivery mechanisms would help to achieve this goal. Protein coronas can shield the surface modifications of nanoparticles, which, however, could be manipulated for specific targeting. Liposomal surfaces modified with peptides derived from Ab1-42 could specifically interact with the lipid-binding domain of exchangeable apolipoproteins to mediate brain-targeted delivery [201]. Biomimetic systems leveraging cells or cell-derived materials endow delivery systems with natural properties such as specific affinity, homing, targeted motility and specific therapeutic effects, e.g., immunomodulation [202,203]. Microrobots or nanorobots containing activatable engines or integrated with biological carriers such as sperm, bacteria, macrophages, neutrophils, red blood cells, and platelets are springing up due to their motility, allowing the active targeting and penetration of tissues [204,205]. Delivery systems should be intelligent enough to target an injured heart, for which strategies integrating the design of materials with biological systems may be helpful.

## Figures and Tables

**Table 1:** Gene therapy in clinical trials to treat heart diseases.

NCT Number	Gene Target	Disease	Administration Route	Delivery Method	Clinical Phase	Current Status	Results	References
NCT01643330	SERCA2a	Advanced heart failure	Percutaneous/intracoronary administration	AAV1 vector	Terminated in phase 2b	Terminated	Safe but no improvement in outcomes in phase 2b	[9,10,11,12,13]
NCT00787059	Adenylyl cyclase type 6 (AC6)	Congestive heart failure	Intracoronary administration	Adenovirus-5 (Ad5)	Phase 2 and FDA approved phase 3	Completed	Improvement in LV function	[14]
NCT02694575	SDF-1	Chronic heart failure	Endocardial injection	Plasmid DNA	Phase 2	Completed	Increased EV and SV at 12 months	[15]
NCT03039751	VEGF-D	Severe coronary heart disease	Endocardial injection	Adenovirus	Phase 2	Recruiting	\	[16,17]
NCT00936819	Endothelial nitric oxide synthase (eNOS)	Acute myocardial infarction (AMI)	Intracoronary injection	Endothelial progenitor cells transfected with linear polyethyleneimine (jetPEI)	Phase 2b	Recruiting	\	[18,19]
NCT00135850	VEGF 165	Ischemic cardiopathy	Intramyocardial injection	Plasmid	Phase 2	Completed	Safe but transient improvement in myocardial perfusion	[20,21]
NCT02928094	FGF-4	Myocardial infarction	Intracoronary administration	Adenovirus-5 (Ad5)	Phase 3	Not yet recruiting	Terminated for ASPIRE; not yet recruiting for AFFIRM	[22,23,24,25]
NCT04125732	VEGF	Refractory angina coronary	Transthoracic epicardial procedure	XC001 (AdVEGFXC1)	Phase 2	Recruiting	\	[26]
NCT04179643	I-1 transgene (AA 1-65 with T35D)	Heart failure	Intracoronary infusion	BNP116 AAV	Phase 1	Recruiting	\	[27]

## Data Availability

Not applicable.
